# Effect of Almond Consumption on Metabolic Risk Factors—Glucose Metabolism, Hyperinsulinemia, Selected Markers of Inflammation: A Randomized Controlled Trial in Adolescents and Young Adults

**DOI:** 10.3389/fnut.2021.668622

**Published:** 2021-06-24

**Authors:** Jagmeet Madan, Sharvari Desai, Panchali Moitra, Sheryl Salis, Shubhada Agashe, Rekha Battalwar, Anushree Mehta, Rachana Kamble, Soumik Kalita, Ajay Gajanan Phatak, Shobha A. Udipi, Rama A. Vaidya, Ashok B. Vaidya

**Affiliations:** ^1^Sir Vithaldas Thackersey College of Home Science (Autonomous), Shreemati Nathibai Damodar Thackersey (SNDT) Women's University, Mumbai, India; ^2^Nurture Health Solutions, Mumbai, India; ^3^Clinical and Endocrine Laboratory, Kasturba Health Society Medical Research Centre, Mumbai, India; ^4^Kasturba Health Society Medical Research Centre, Mumbai, India; ^5^FamPhy, Gurgaon, India; ^6^Charutar Arogya Mandal, Karamsad, India; ^7^Department of Food Science and Nutrition, Shreemati Nathibai Damodar Thackersey (SNDT) Women's University, Mumbai, India; ^8^Division of Endocrine and Metabolic Disorders, Kasturba Health Society Medical Research Centre, Mumbai, India

**Keywords:** almonds, prediabetes, IL-6, LDL-C, HbA1c, hyperinsulinemia

## Abstract

A large percentage of the Indian population has diabetes or is at risk of pre-diabetes. Almond consumption has shown benefits on cardiometabolic risk factors in adults. This study explored the effect of almond consumption on determinants of metabolic dysfunction—blood glucose, lipids, insulin and selected inflammatory markers in adolescents and young adults aged 16–25 years from Mumbai city. This randomized controlled trial was conducted for a period of 90 days on individuals with impaired levels of fasting glucose levels between 100–125 mg/dL (5.6–6.9 mmol/L) and 2-h post-glucose value 140–199 mg/dL (7.8–11.0 mmol/L) and/or fasting insulin (≥15 mIU/ml)/stimulated insulin (≥80 mIU/ml). Of 1,313 individuals screened, 421 met the inclusion criteria, of which 275 consented to participate and 219 completed the trial. The trial was registered with Clinical Trials Registry India (CTRI) CTRI/2018/02/011927. The almonds group (*n* = 107) consumed 56 g almonds daily, the control group (*n* = 112) was provided an iso-caloric cereal-pulse based snack. At baseline and endline, blood glucose, insulin, HbA_1_c, LDL-c, HDL-c, total and ox-cholesterol, triglycerides, hs-CRP, IL-6, TNF-α, adiponectin, leptin were measured and HOMA-IR and FG:FI ratios were calculated. Dietary intakes were assessed. The anthropometric measurements, biochemical markers as well as macronutrient intakes did not differ significantly between the two groups at baseline. Almond consumption significantly decreased HbA_1_c, total cholesterol and LDL-c. Stimulated insulin decreased post-intervention in both groups, but the decrease was greater in the almonds group. Fasting glucose was reduced post intervention in the controls with no change in the almonds group. FG:FI ratio decreased in the almonds group. TNF-α and IL-6 decreased in the almonds group, while it increased in the control group. Our results showed that almonds reduced HbA1c, LDL-c and total cholesterol levels in just 12 weeks of consumption in these adolescents and young adults who were at risk for developing diabetes. Almonds can be considered as part of food-based strategies for preventing pre-diabetes.

**Clinical Trial Registration:** ClinicalTrials.gov, identifier: CTRI/2018/02/011927.

## Introduction

The International Diabetes Federation (1) estimates that the number of people with diabetes is likely to be 153 million in the next two decades (1). According to a large study done on 57,117 individuals above 20 years of age in 14 states of India, the prevalence of diabetes in India was 7.3% whereas that of prediabetes was 10.3% (2). India presently ranks, fourth in the world for the number of adults (20–79 years of age) with impaired glucose tolerance and by 2,045 it is predicted that it will rank third in the world (1). In a large study comprising 1,519 boys and girls in the age group of 6–19 years in an urban center in South India, the prevalence of glucose intolerance was found to be 3.7% with an increase from 4.2 to 12.7% in girls who were found to have abdominal obesity (3). The fact that India has a young population and children in the age group of 6–19 years comprising nearly 30% of the 1.3 billion Indians, the number of children with glucose intolerance is expected to be very large. Indians have the highest per annum progression from pre-diabetes to type 2 diabetes which is around 14–18% (4–8).

Indians in comparison to their Caucasian counterparts have higher body fat as well as visceral fat percentages at similar BMIs which is characterized by the “thin-fat” Indian diabetes phenotype. This particular phenotype may lead to an early onset of diabetes mellitus and metabolic syndrome in Indians in comparison to their Caucasian counterparts (9). Lack of physical activity and unhealthy dietary choices are the major drivers for pre-diabetes. Lifestyle intervention which includes nutritional interventions and physical activity targeted at adolescents as well as young adults is important to help halt the progression from pre-diabetes to type 2 diabetes.

The nutrition transition in India and the replacement of whole grains and traditional wholesome diets with refined carbohydrate, sugar, energy dense, and nutrient poor foods has contributed considerably to this problem (10–12). In a study conducted among 1,026 adolescents, it was found that 70% of the participants reported the consumption of 3 or more servings of energy-dense snacks and 47% reported the consumption of 3 or more servings of energy-dense drinks in a single day (13). There are no studies that have looked at targeted nutritional interventions for adolescents or young adults in India, where snacking is a very common phenomenon and therefore there is a need to explore the same to help them have healthy snacking choices. Tree nuts with their unique composition offer a natural choice as a whole food that can be included by persons prone to poor metabolic health (14, 15). They represent one of the healthiest snacking options as a food-based strategy to achieve better metabolic health in terms of insulin sensitivity, reduced inflammation, and lipid profile (16). Almonds alone or in combination with carbohydrate rich foods have been found to significantly reduce postprandial glucose, insulin response, fasting glucose, and glycosylated hemoglobin in adults (16–20). Most of the studies looking at the effect of almonds on glucose metabolism have been done in the western population, few studies have been conducted in Asian Indians and none have been conducted on adolescents and young Indian adults who have a proclivity for insulin resistance and Type 2 diabetes. In this context, the present randomized controlled trial was undertaken to examine the effect of almond consumption on glucose metabolism, hyperinsulinemia, selected markers of inflammation, and lipid profile in young adults and adolescents residing in urban Mumbai, India.

## Materials and Methods

### Study Design

This was a randomized controlled, open-label, parallel arm study conducted on community living adolescents and young adults (16–25 years of age) in Mumbai, India. The inclusion criteria were participants in the age group of 16–25 years of age, with impaired fasting glucose levels between 100 and 125 mg/dL (5.6–6.9 mmol/L) and 2-h post-glucose value 140–199 mg/dL (7.8–11.0 mmol/L) (21) and/or fasting hyperinsulinemia (≥15 mIU/ml) or glucose challenge hyperinsulinemia (≥80 m IU/ml) (22, 23). The exclusion criteria included the presence of any known chronic disease, known history of food allergies with nuts, on prescribed medications like steroids, state of pregnancy and/or lactation.

The study was conducted from September 2017 to February 2019.

The study was approved by the Intersystem Biomedical Ethics Committee, Mumbai, India (ISBEC version 2 dated 12th August, 2017) and conducted according to Good Clinical Practices and the Declaration of Helsinki. Informed written consent was taken from each institution, each participant and each guardian/parent for participants who were between 16 and 18 years of age; both before screening and prior to participant enrolment for the trial.

The trial was registered with Clinical Trials Registry India (CTRI) CTRI/2018/02/011927 (24) and therefore has been registered in the International Clinical Trials Registry Platform (ICTRP) (25).

**Figure 1:**
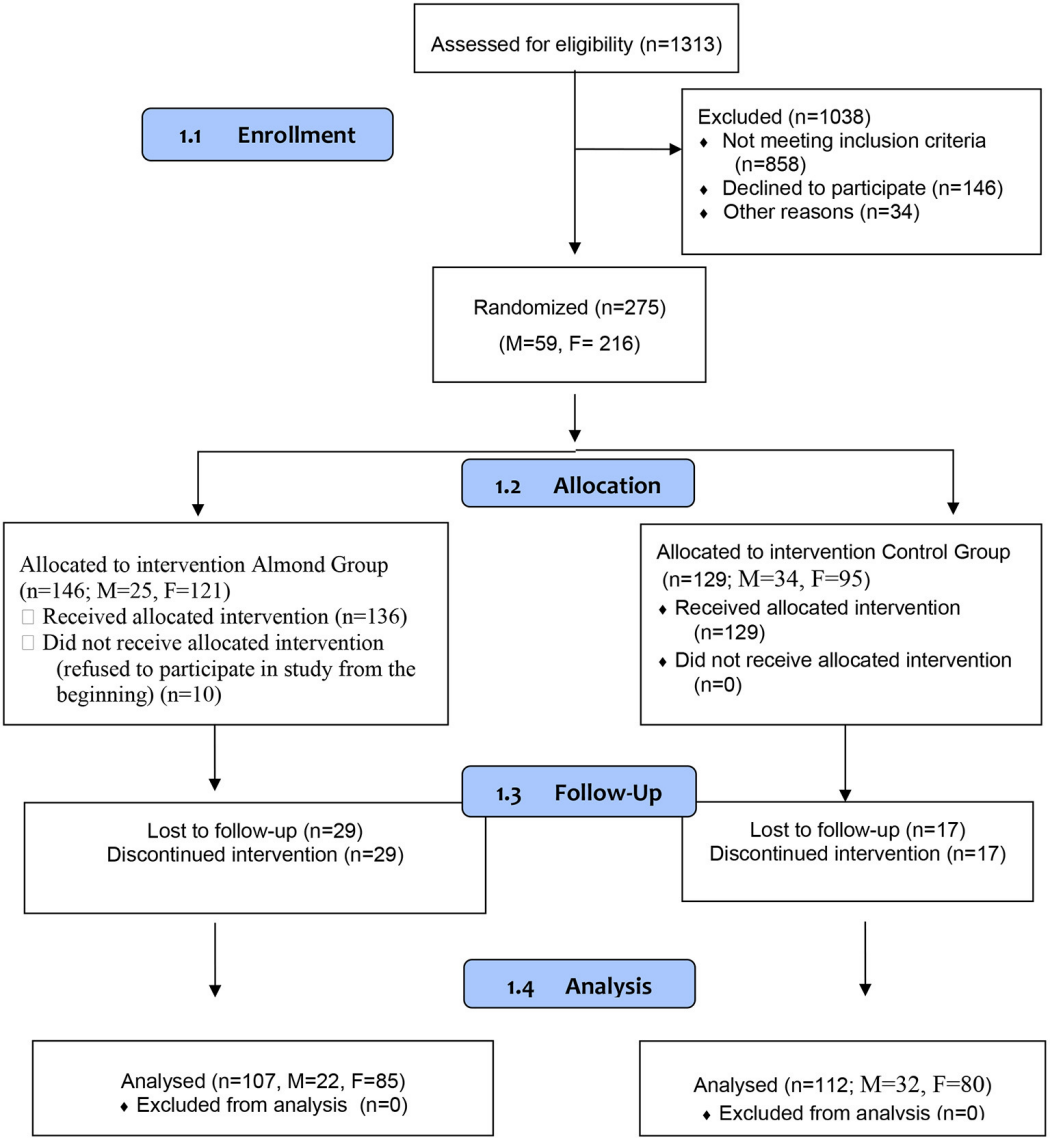
Study design.

Sample size estimation: Fasting blood glucose was considered a primary outcome for sample size calculations. In the absence of reliable regional data, an effect size of 0.4 was assumed. With this effect size, a sample of about 112 participants per group was required allowing for 5% alpha (type I) error and to achieve 85% power. Assuming 20% dropouts, an evaluable sample size of about 270 participants was required. The design of the trial is illustrated in Figure 1.

Participants were those attending various academic institutions in Mumbai city. Twenty-four academic institutions in Mumbai city were contacted and the study objectives and protocol were explained to the administration/authorities. Of these, 11 provided their consent to recruit and conduct the study. All consenting participants (*n* = 1,313) gave blood samples for measurement of fasting glucose as well as stimulated glucose (2 h after consumption of 75 g of glucose) and fasting, stimulated insulin. Anthropometric measurements were also recorded for each participant during this time.

### Randomization and Treatment

Among the 1,313 participants who were screened, 421 participants (males 88, females 333) were eligible because they had elevated fasting/stimulated blood glucose [fasting glucose (100–125 mg/dl), 2h post-glucose (140–199 mg/dl)] and/or insulin [fasting insulin (≥15mIU/ml)/stimulated insulin (≥80mIU/ml)]. Among these 421 individuals, 146 did not provide consent to participate in the intervention. Thus, 275 consenting individuals (males 59, females 216) participated in the study. Baseline assessment was done for fasting blood glucose, fasting insulin, stimulated (2-h post 75 g glucose administration) blood glucose, stimulated insulin, glycosylated hemoglobin (HbA1c), lipid profile, oxidized LDL, adiponectin, leptin, inflammatory markers -IL-6 and TNF-α. Cluster randomization within each institution was used to then assign the individuals either to the almonds group or the control group. Almonds were supplied by the Almonds Board of California, USA. They were stored at 10–15°C in sealed polyethylene bags which were then hygienically packaged and sealed in cardboard cartons. The participation by females was higher as compared to males in the study.

### Intervention

Participants in the almonds group received 56 g of raw almonds daily (providing ~20% of the daily energy intake i.e., 340 kcals/d) which was distributed in 2 packets that were to be consumed as snacks in a day. Each day's supply was packaged individually in polyethylene re-sealable bags.

Participants in the control group received commonly consumed isocaloric (as provided by 56 g of raw almonds) Indian savory snacks of 2 varieties to prevent taste fatigue. The control snack was prepared using whole wheat flour, chickpea flour, salt, and Indian spices (nutrient composition of control snack is provided in Supplementary Table 1). The snack was selected based on a pre-screening survey of snacking patterns in the age group of the study subjects. The control snacks were packaged in laminated sealed pouches. The nutritional composition of the control snacks was compared to those of the almonds provided to the intervention group to decide on the portion size and on average the control snack weighed 61–65 g. The entire week's supply for the almonds or the control snacks was provided twice a week to the participants of the respective groups. Participants were instructed to continue with their regular diets and lifestyle including exercise patterns. Weekly monitoring was done for each participant by the supervising physician for any adverse effects.

### Measurements

Each participant underwent a clinical examination that was conducted by a physician to assess the general health status at screening, baseline as well as at the end of the study. Anthropometric measurements i.e., weight, height, waist circumference, and hip circumference were assessed by trained research assistants using valid and established procedures ensuring that they wore light clothing and no shoes. Body composition was measured using a TANITA body composition analyzer (Model MC 780 MA).

### Biological Samples, Collection, Storage, and Biochemical Measurements

Participants were asked to report after fasting overnight for at least 12 h. At time points indicated under methodology, a trained phlebotomist collected fasting blood samples at each individual site/institute. Whole blood was analyzed for complete blood count including hemoglobin, White Blood Cells (WBC), Red Blood Cells (RBC), platelets, Mean Corpuscular Volume (MCV), Mean Corpuscular Hemoglobin (MCH), and Mean Corpuscular Hemoglobin Concentration (MCHC). Serum was separated by centrifugation, divided into aliquots and stored at −70°C until analyses.

Glucose tolerance test (2-h post 75-g glucose administration) was conducted at screening, at baseline and at the end of the study; glucose was measured spectrophotometrically by the Glucose oxidase (GOD), Peroxidase (POD), method (Accurex Biomedical Pvt Ltd.).

Insulin was measured by radioimmunoassay using a Beckman Coulter Counter. Glycosylated hemoglobin (HbA1c) was measured using Nycocard reader (Alere Technologies, Norway). HOMA-IR was calculated according to the formula: fasting serum insulin (μU/ml) × fasting plasma glucose (mmol/l)/22.5. The fasting glucose to fasting insulin ratio (FG:FI) was also calculated.

Lipid profile was also measured at baseline and end of the study using kits: cholesterol (Accurex Biomedical Pvt Ltd.), triglycerides (Accurex Biomedical Pvt Ltd.), High density lipoprotein cholesterol (HDL-c) (Coral Clinical Systems) were measured. Low density lipoprotein cholesterol (LDL-c) was calculated using the Friedewald formula (26) in those subjects whose triglyceride levels were <400 mg/dl. Oxidized LDL was measured by ELISA method with kits from ELab Science Biotechnology Inc., USA. Adiponectin, leptin, TNF-α and IL-6 were measured by the ELISA kits provided by DIA source, Belgium.

### Compliance to Intervention

Each participant was contacted over the phone every 2 days to check for the consumption of either almonds or the control product. Participants were instructed to return the unused portions twice a week that were weighed to estimate the intake during a 7-day period.

### Dietary Assessment

At baseline and the end of the intervention, dietary intakes were estimated by trained research assistants using 1 day 24-h diet recall. Standard measuring cups, glasses, spoons, as well as food models, were used to improve the accuracy of estimation. Nutrient analysis of the dietary data was done using “DietCal” Version 8.0 (27) software by Profound Tech Solutions which is based on values from Indian Food Composition Tables 2017.

### Statistical Analyses

Descriptive statistics [Mean ± SD, Frequency (%)] were used to present the socio-demographic and baseline clinical profile of the participants. Paired-*t* test was used to assess the impact of the intervention (if any) from baseline to endline, within each group. To adjust for any baseline differences, an independent sample *t*-test on difference (delta) was used to compare the magnitude of the impact across groups. Both intent-to-treat (ITT) and per-protocol (PP) approaches were used for statistical analysis. ITT analysis included all randomized participants for whom baseline data were available. The PP analysis excluded those participants who did not attend the last visit and for whom endline data were not available or who consumed <80% of the almonds/control snacks. The data indicated no difference between participants who completed the study protocol and who discontinued the study/ lost to follow up. Albeit the variables involved were checked for normality, considering the large sample size; parametric tests were performed as suggested by Norman (28). The analysis was performed using STATA (14.2). Significance was defined as *p* < 0.05.

## Results

The baseline characteristics of participants in the almond group did not differ significantly from those in the control group for any of the anthropometric and biochemical measurements. There was no significant change observed post-intervention in anthropometric measurements as well as body composition, within each group in comparison to baseline.

Table 1 shows the changes after intervention in the anthropometry and biochemical parameters in comparison to baseline in both groups.

**Table 1:** Mean baseline and change in anthropometric indices, glucose, insulin, lipid profile, inflammatory markers, and oxidized LDL in control and almonds groups.

**Characteristics**	**Almond group (*****n*** **=** **107) Mean** **±** **SD (95% CI)**		**Control group (*****n*** **=** **112) Mean** **±** **SD (95% CI)**		**Treatment effect *P***
	**Baseline**	**Change**	**Baseline**	**Change**	
Weight (kg)	59.6 ± 15.1 (56.7, 62.5)	0.92 ± 1.65 (0.60, 1.24)	56.6 ± 13.6 (54.0, 59.1)	0.52 ± 4.17 (−0.25, 1.30)	0.35
BMI (kg/m^2^)	23.7 ± 5.4 (22.67, 24.72)	0.35 ± 0.66 (0.22, 0.47)	22.4 ± 4.8 (21.55, 23.34)	0.19 ± 1.77 (−0.13, 0.53)	0.40
Waist-to-hip ratio	0.79 ± 0.08 (0.78, 0.81)	0.01 ± 0.05 (0.00, 0.02)	0.80 ± 0.06 (0.78, 0.81)	0.00 ± 0.0 (−0.00, 0.10)	0.11
Waist-to-height ratio	0.47 ± 0.08 (0.46, 0.49)	0.01 ± 0.03 (0.00, 0.01)	0.46 ± 0.07 (0.45–0.47)	0.01 ± 0.03 (0.00, 0.01)	0.94
Percent body fat (%)	30.3 ± 8.7 (29.3, 32.6)	0.87 ± 2.12 (0.46, 1.27)	27.5 ± 8.5 (26.3, 29.6)	3.26 ± 18.07 (−0.12, 6.64)	0.17
Visceral fat	5.02 ± 3.45 (4.34, 5.70)	0.11 ± 0.94 (−0.07, 0.29)	4.29 ± 3.24 (3.66, 4.93)	0.12 ± 1.08 (−0.08, 0.34)	0.91
Fasting glucose (mg/dL)	80.62 ± 7.14 (79.16, 81.93)	0.02 ± 12.0 (−2.27, 2.31)	84.90 ± 11.16 (82.60, 86.68)	−5.5 ± 14.6 (−8.24, −2.75)	0.01
2 h glucose (mg/dL)	102.97 ± 21.41 (98.54, 106.72)	−8.3 ± 22.3 (−12.59, −4.03)	106.84 ± 23.17 (102.14, 110.77)	−12.1 ± 26.5 (−17.05, −7.12)	0.25
Fasting insulin (mIU/L)	11.24 ± 6.34 (10.03, 12.46)	1.6 ± 13.4 (−0.93, 4.18)	11.60 ± 5.36 (10.60, 12.63)	−0.3 ± 5.5 (−1.35, 0.68)	0.15
Stimulated insulin (mIU/L)	138.42 ± 78.60 (124.46, 154.50)	−29.7 ± 83.7 (−45.7, −13.6)	121.77 ± 52.91 (111.82, 131.73)	−20.3 ± 78.3 (−35, −5.6)	0.39
HbA1c (%)	5.38 ± 0.35 (5.35, 5.55)	−0.04 ± 0.44 (−0.12, 0.04)	5.33 ± 0.27 (5.27, 5.38)	0.09 ± 0.40 (0.01, 0.16)	0.02
HOMA-IR	2.25 ± 1.34 (1.99, 2.51)	0.61 ± 5.53 (−0.44, 1.67)	2.45 ± 1.25 (2.21, 2.68)	−0.19 ± 1.29 (−0.43, 0.04)	0.13
FG:FI	9.04 ± 4.22 (8.22, 9.85)	−0.55 ± 4.22 (−1.35, 0.26)	9.00 ± 4.82 (8.09, 9.90)	0.22 ± 5.49 (−0.80, 1.25)	0.24
Total cholesterol (mg/dL)	151.40 ± 31.19 (145.76, 157.74)	−5.70 ± 24.63 (−10.42, −0.98)	138.46 ± 23.73 (134.05, 142.93)	13.35 ± 94.74 (−4.38, 31.09)	0.04
Triglycerides (mg/dL)	75.54 ± 37.99 (68.37, 83.06)	−2.74 ± 34.87 (−9.42, 3.94)	75.12 ± 35.53 (68.47, 81.77)	1.7 ± 31.81 (−4.25, 7.65)	0.32
HDL-c (mg/dL)	43.60 ± 14.39 (40.84, 46.37)	−1.07 ± 13.25 (−3.61, 1.46)	42.66 ± 9.58 (40.91, 44.50)	−0.55 ± 9.07 (−2.25, 1.13)	0.73
LDL-c (mg/dL)	93.33 ± 28.04 (87.93, 98.73)	−4.27 ± 24.85 (−9.05, 0.51)	80.68 ± 21.10 (76.74, 84.64)	5.93 ± 21.26 (1.95, 9.91)	0.01
VLDL-c (mg/dL)	15.27 ± 7.81 (13.77, 16.79)	−0.70 ± 7.21 (−2.08, 0.68)	15.01 ± 7.12 (13.67, 16.34)	0.35 ± 6.38 (−0.84, 1.54)	0.25
hs-CRP (mg/L)	3.94 ± 6.52 (2.69, 5.19)	0.17 ± 6.16 (−1.01, 1.35)	2.75 ± 4.11 (1.98, 3.52)	0.04 ± 4.73 (−0.84, 0.92)	0.85
Adiponectin (μg/mL)	7.23 ± 4.06 (6.43, 8.00)	−0.04 ± 1.93 (−0.41, 0.32)	7.90 ± 4.06 (7.14, 8.66)	0.03 ± 2.28 (−0.39, 0.45)	0.78
Leptin (ng/mL)	13.17 ± 7.86 (11.50, 14.43)	1.28 ± 4.21 (0.47, 2.09)	11.22 ± 7.93 (9.74, 12.71)	0.67 ± 3.47 (0.02, 1.33)	0.24
IL-6 (pg/ml)	61.54 ± 192.87 (24.81, 99.42)	−36.12 ± 188.60 (−72.27, 0.02)	35.34 ± 62.34 (23.68, 47.02)	−2.25 ± 58.59 (−13.22, 8.71)	0.07
TNF Alpha (pg/ml)	22.17 ± 30.68 (16.28, 28.15)	−0.28 ± 38.06 (−7.57, 7.01)	17.21 ± 22.52 (13.00, 21.44)	2.64 ± 41.09 (−5.04, 10.34)	0.58
Ox LDL (pg/ml)	249,076 ± 72,406 (235,139, 262,768)	12218.9 ± 57935.7 (1,115, 23,323)	234,870 ± 66,163 (222,481, 247,258)	7012.6 ± 46855.5 (−1,760, 15,786)	0.46

*All values are means (95% CI). Intragroup analysis was assessed by the paired Student t-test. Significant difference (p, 0.05) between baseline and end of an intervention period*.

### Effect of Intervention on Glucose Metabolism

The mean glycosylated hemoglobin levels (HbA1c) levels showed a significant reduction in the almond group in comparison to the control group (Table 1). There was no significant difference in HOMA-IR at the end of the study compared to baseline between the two groups and within each group. There was a decrease in the fasting blood glucose to fasting insulin ratio (FG:FI) in the almond group in comparison to the control group but was not statistically significant. The fasting blood glucose levels were significantly reduced in the control as compared to the almond group. The other biomarkers for glucose metabolism showed no significant difference between the almond and the control groups at the end of the study in comparison to baseline (Table 1).

### Effect of Intervention on Lipid Profile

There was a significant reduction in the total cholesterol and LDL-c levels in the almond group in comparison to the control group. There was an increase in the HDL-c levels, a decrease in the triglyceride levels as well as a decrease in the VLDL-c levels in the almond group in comparison to the control group but was not statistically significant (Table 1).

### Effect of Intervention on Inflammatory Markers

There was a decrease in the inflammatory marker, IL-6 in the almond group in comparison the control group which was not statistically significant (*p* = 0.07) (Table 1).

### Tolerability

The participants were monitored by the supervising physicians to observe if they had any gastro-intestinal disturbances during the study. In the almonds group, one participant complained of transient gastric irritation and one had diarrhea, another participant had skin eruptions and both were withdrawn from the study. There was another participant who complained about a rash on cheeks, itching and a sense of heaviness. This participant was also withdrawn from the study and was treated with an anti-allergic medication for 5 days which managed his symptoms. His blood sample was tested for IgE level which was 159 IU/ml which confirmed it was an allergic reaction. The participant was followed up for a month and was observed to be asymptomatic. All the adverse events were reported to the Ethics Committee.

## Discussion

Consumption of unhealthy snacks made from refined foods has increased in India, often replacing meals and traditional whole foods, resulting in compromised nutrient intakes (29). This may unfavorably influence metabolic health and increase the risk for the development of obesity as well as obesity associated non-communicable diseases (NCDs). One of the possible ways to improve metabolic health and reduce the risk of NCDs is by introducing healthy snacks (30). Snacking is a common phenomenon in India especially in the young. This study looked at a healthy alternative snack that can help replace unhealthy snacks, particularly in the Indian market. Hectic lifestyle especially in the young population makes them reach out for snacks and therefore it is important to find healthy alternatives to popular snacks. The present study looked at whether almonds could influence glycemic and lipid markers if it is consumed as a replacement for popular snacks, in this part of the world.

Almonds have the potential not only to replace unhealthy snacks but have been shown to improve several metabolic parameters. Several researchers have studied the effect of almond intake on blood glucose and insulin levels (31–35). There has been no study done to look for the effects of almond consumption on different parameters of metabolic health in adolescents and young adults who are predisposed to diabetes. This study has been able to demonstrate the effect of almonds on reducing important parameters of glucose metabolism like HbA1c which is a measure of the average blood glucose levels over the past 2–3 months. The results of this randomized controlled clinical trial showed that almonds have the potential to reduce hyperinsulinemia to help improve insulin resistance prior to pre-diabetes stage. Almonds have been shown to influence insulin and HOMA IR. The monounsaturated fatty acid content of almonds may play a favorable role in insulin resistance (34, 36).

Although fasting glucose is used in clinical settings to assess metabolic health, it has limitations as a biomarker. HbA1c measurement is a more reliable marker than either fasting or post prandial blood glucose levels. It not only equals the assessment of hundreds (virtually thousands) of fasting glucose levels but also captures postprandial glucose peak which is an important marker for insulin resistance (37). Most of the participants in the present study did not have either impaired fasting or high post prandial glucose levels and were included in the study as they were found to be insulin resistant. This may be regarded as one of the strengths of the present study, where participants were studied at an earlier stage of disturbance in metabolic health, much before blood glucose levels became elevated.

Several studies in literature have reported that almonds reduced LDL-c, non HDL-c, and central adiposity while maintaining HDL-c concentrations (38–44). A similar trend was observed in the present study with this young age group wherein, LDL-c levels were significantly reduced in the almonds group. The total cholesterol levels were also reduced significantly in the almond group. In a recent systematic review on tree nut, Altamimi et al. (45) reported that almonds lowered LDL- c and that effects were observed with the consumption of even 20 g of the nut. The beneficial effects of almonds could be attributed to the fact that our participants were asked to consume raw almonds with skin. Also, almonds contain a significant amount of monounsaturated fatty acids (46). A study reported that flow-mediated dilation and soluble vascular cell adhesion molecules were reduced in overweight persons with the intake of almonds wherein, the almond diet provided 7.6% of energy from polyunsaturated fatty acids that have a beneficial effect in terms of reducing LDL-c and either maintaining or increasing HDL-c (47). Almonds have also been found to have good antioxidant capacity and to protect DNA from oxidative damage (48). Studies have shown that the consumption of almonds helped reduce inflammatory markers like IL-6 (49, 50). In the present study there was a reduction in inflammatory markers like IL-6, but it did not attain the statistical level of significance (*p* = 0.07).

Snacks like cookies, muffins, bars etc. that are popular in this age group could lead to a significant increase in blood glucose and insulin, followed by a rapid drop in blood glucose because of the increase in insulin levels, leading to the individual feeling hungry again (35, 38, 43, 51). The snack given to the control group in the present study was carefully planned to ensure that it contained complex carbohydrates and no added sugar. One of the strengths of the present investigation is that a younger age group was studied without a change in their lifestyle or diet, indicating the potential for preventing progression into further metabolic dysfunction precipitating them to risk of prediabetes and further into the development of Type 2 diabetes. The intervention was the inclusion of almonds versus the control snack eaten in two divided doses rather than the substitution of a snack in their daily meals. We believe therefore that the intervention effected the quality of the diet through addition of almonds in a defined amount per day. Thus, the inclusion of almonds in a balanced diet has the potential to be a nutritious natural food- based strategy rather than use of a nutraceutical to help reverse the risk for prediabetes. However, it may be worthwhile to conduct a long-term study that would examine the effect of almonds along with dietary counseling and/or physical exercise.

A limitation of this study similar to many food-based trials is that participants could not be blinded, although clustered randomization was done to overcome this and to avoid contamination between groups. Further, a nutritional intervention study can also trigger behavioral changes both in the intervention group as well as in the control group as participants are made aware of aware of their risk during the recruitment process, therefore became more conscious about their health and the lifestyle modification to be made.

Extraneous variables such as culturally relevant periodic fasting which is not uncommon may have also had some influence on the outcome of the trial.

In an incidental finding, participants who had reported having acne at the start of the trial reported better skin texture after the trial, especially in the almond group. Although acne was not studied as an outcome in this study, it can be a subject of a future trial in adolescents and young adults as it has been shown to correlate with metabolic syndrome (52). There is a close interplay between metabolic and hemodynamic derangements that include oxidative stress, inflammation and advanced glycation end products that are involved in the etiopathogenesis of poor metabolic health, prediabetes and finally Type 2 diabetes.

Overall, in the present study, almonds were shown to have an effect on glucose metabolism by reducing HbA1c levels in just 12 weeks of consumption in adolescents and young adults. The study has also shown that almonds when included as a snack can help manage dyslipidemia by reducing LDL-c and total cholesterol in the same population. Almonds can be a good healthy snack that can replace regular snacks and can be considered as a part of the food-based strategy to help prevent prediabetes especially in the young.

## Data Availability Statement

The original contributions presented in the study are included in the article/Supplementary Materials, further inquiries can be directed to the corresponding author.

## Ethics Statement

The studies involving human participants were reviewed and approved by Intersystem Biomedical Ethics Committee, Mumbai, India (ISBEC version 2 dated 12th August, 2017). Written informed consent to participate in this study was provided by the participants' legal guardian/next of kin.

## Author's Note

An abstract of this article was selected as an oral communication at the Nutrition 2020 Live Online—a symposium of American Society of Nutrition 2020 held on June 1st to June 4th 2020, USA.

## Author Contributions

JM, RV, SU, and AV were responsible for the design of this clinical trial, obtaining funding, and executing it. JM was a PI, RV and SU were co PI's who contributed to writing of paper. SK oversaw the execution of study and critically revised the manuscript for important content. SD was the data manager and SA did all the laboratory analysis. PM, SS, and RB were coordinators for day to day implementation, data collection, and recruitment of subjects from different institutions. AM and RK were the physicians responsible for supervising and monitoring health of the participants throughout the trial. AP was the statistician and analyzed the data. All authors provided administrative, technical, or material support. JM, RV, SU, AV, and SK had full access to all the data in the study and take responsibility for the integrity of the data and the accuracy of the data analysis.

## Conflict of Interest

The authors declare that the research was conducted in the absence of any commercial or financial relationships that could be construed as a potential conflict of interest.
