# Supplementary material for: Effect of Almond Consumption on Metabolic Risk Factors—Glucose Metabolism, Hyperinsulinemia, Selected Markers of Inflammation: A Randomized Controlled Trial in Adolescents and Young Adults
Source: Front Nutr. 2021 Jun 24;8:668622. doi: 10.3389/fnut.2021.668622 (PMC8264510; doi:10.3389/fnut.2021.668622)
Supplement: Supplementary file 1 [file Table_1.DOC]

**SUPPLEMENTARY TABLE 1: NUTRIENT COMOSITION OF EXPERIMENTAL AND CONTROL SNACK**

| **NUTRIENT COMPOSITION** | **INTERVENTION GROUP** | **CONTROL SNACKS** | |
| --- | --- | --- | --- |
|  | **Raw Almonds**  **(56g)** | **Variety 1**  **(61g)** | **Variety 2**  **(64g)** |
| **Energy (kcals/day)** | 320 | 339 | 343 |
| **Total Carbohydrates (grams)** | 12.0 | 26.0 | 27.1 |
| **Proteins**  **(grams)** | 12.0 | 7.3 | 7.6 |
| **Fats**  **(grams)** | 28.0 | 22.5 | 22.6 |

**Note:**

The snack was based on a combination of whole grain wheat and chickpea flour. It contained only complex carbohydrates and did not contain any free or added sugar.
